# Linalool oxide: generalist plant based lure for mosquito disease vectors

**DOI:** 10.1186/s13071-015-1184-8

**Published:** 2015-11-09

**Authors:** Vincent O. Nyasembe, David P. Tchouassi, Charles M. Mbogo, Catherine L. Sole, Christian Pirk, Baldwyn Torto

**Affiliations:** Behavioral and Chemical Ecology Department, International Centre of Insect Physiology and Ecology, P.O Box 30772–00100, Nairobi, Kenya; KEMRI & Public Health Department, Centre for Geographic Medicine Research – Coast, KEMRI – Wellcome Trust Research Programme, Nairobi, Kenya; Department of Zoology and Entomology, University of Pretoria, Pretoria, South Africa

**Keywords:** Rift Valley fever, Dengue, Climate-change, Stereoisomers, Arboviral diseases, Odor-bait, Vector ecology, *Aedes mcintoshi*, *Aedes ochraceus*, *Aedes aegypti*

## Abstract

**Background:**

Lack of effective vaccines and therapeutics for important arboviral diseases such as Rift Valley fever (RVF) and dengue, necessitates continuous monitoring of vector populations for infections in them. Plant-based lures as surveillance tools has the potential of targeting mosquitoes of both sexes and females of varied physiological states; yet such lures are lacking for vectors of these diseases. Here, we present evidence of the effectiveness of linalool oxide (LO), a single plant-based lure previously developed for malaria vectors in trapping RVF vectors, *Aedes mcintoshi* and *Aedes ochraceus*, and dengue vector, *Aedes aegypti*.

**Methods:**

For RVF vectors, we used CDC traps to evaluate the performance of LO against three vertebrate-based lures: CO_2_ (dry ice), BioGent (BG) lure, and HONAD (a blend of aldehydes) in 2 experiments with Completely Randomized design: 1) using unlit CDC traps baited separately with LO, HONAD and BG-lure, and unlit CDC trap + CO_2_ and lit CDC trap as controls, 2) similar treatments but with inclusion of CO_2_ to all the traps. For dengue vectors, LO was evaluated against BG lure using BG sentinel traps, in a 3 × 6 Latin Square design, first as single lures and then combined with CO_2_ and traps baited with CO_2_ included as controls. Trap captures were compared between the treatments using Chi square and GLM.

**Results:**

Low captures of RVF vectors were recorded for all lures in the absence of CO_2_ with no significant difference between them. When combined with CO_2_, LO performance in trapping these vectors was comparable to BG-lure and HONAD but it was less effective than the lit CDC trap. In the absence of CO_2_, LO performed comparably with the BG-lure in trapping female *Ae. aegypti*, but with significantly higher males recorded in traps baited with the plant-based lure. When CO_2_ was added, LO was significantly better than the BG-lure with a 2.8- fold increase in captures of male *Ae. aegypti*.

**Conclusions:**

These results highlight the potential of LO as a generalist plant-based lure for mosquito disease vectors, pending further assessment of possible specificity in their response profile to the different stereoisomers of this compound.

**Electronic supplementary material:**

The online version of this article (doi:10.1186/s13071-015-1184-8) contains supplementary material, which is available to authorized users.

## Background

Vector-borne diseases exert a huge toll on global infectious disease burden. Rift Valley fever (RVF) and dengue represent two important mosquito-borne arboviral diseases, which continue to spread, evident from numerous disease outbreaks in various parts of the world [1, 2]. Rift Valley fever is an epizootic disease mainly occurring in Africa and the Arabian Peninsula, with outbreaks leading to devastating loss of millions of livestock and thousands of human deaths [3–7]. On the other hand, dengue, which mainly affects humans, has a worldwide distribution where outbreaks have been reported in over 110 countries [8]. Approximately 3 billion of the world population is at risk of dengue infection and over 100 million reported cases and up to 25000 fatalities annually [1, 9]. Both RVF and dengue have episodic outbreak patterns with low viral activities during the inter-epidemic periods [2, 10, 11]. Rift Valley fever outbreaks are associated with weather anomalies such as widespread elevated rainfall while that of dengue is closely linked to urbanization and transportation, which creates conducive breeding sites for the respective vector populations, and subsequent virus amplification and transmission [12–14]. In the recent past, there has been a growing concern of the possibility of further spread of both diseases to new areas, particularly to Asia and Europe, in the wake of current climate change [14, 15]. In Kenya, the key primary vectors implicated in the transmission of RVF are the flood water *Aedes mcintoshi* and *Aedes ochraceus* [16, 17] while that of dengue fever is *Aedes aegypti* [18]. Lack of safe and effective vaccines and therapeutics against both diseases [1, 19], makes studies on the vectors geared towards developing efficient monitoring or control tools a priority [12, 20]. Effective monitoring of infection/viruses in vectors requires highly effective sampling tools.

The successful use of odor bait technology in population reduction of *Glossina morsitans morsitans* Westwood and *G. pallidipes* Austen in the Zambezi Valley of Zimbabwe [21], has heightened prospects for its similar application in the surveillance and control of blood feeding insects [22]. For blood feeding mosquitoes, a number of odor baits targeting specific species have been developed with considerable success, but these baits are mainly based on vertebrate host odors [23–26]. These baits have been widely employed along with carbon dioxide, which is associated with vertebrate breath and is known to elicit long range activation of host seeking behavior in most mosquito species [27]. In addition, the synthetic carbon dioxide that is extensively employed together with these lures is expensive and presents logistical challenges for use in remote areas where these diseases are endemic [28]. These challenges can be circumvented by employing plant based lures as adult mosquitoes of all physiological states and both sexes utilize nectar for energy [29, 30]. However, except for a few laboratory studies to identify plant odors attractive to *Ae. aegypti* [30], little effort has been made to develop plant based lures for the management of RVF and dengue vectors.

In an effort to develop more potent lures for these vector species, we investigated the potential of linalool oxide (LO), a single-component plant based lure initially developed for the malaria vectors [31, 32], in trapping the primary vectors for RVF, *Ae. mcintoshi* and *Ae. ochraceus*; and dengue fever, *Ae. aegypti*. This study presents the first evidence of effectiveness of a plant-based lure in trapping primary RVF and dengue vectors.

## Methods

### Study sites

The study was conducted at two sites in Kenya: Garissa County in North Eastern region where RVF is endemic [17] and Kilifi County in Coastal region where dengue fever is endemic [33]. The two sites were selected based on the relative abundance of the target vectors of these diseases i.e. *Aedes* (*Neomelaniconion*) *mcintoshi* and *Aedes* (*Aedimorphus*) *ochraceus* for RVF, and *Aedes aegypti* for dengue [25, 34, 35].

Garissa County is largely semi-arid with two unreliable rainy seasons a year; short rains occurring between October and December and the long rains between March and May. Typical average rainfall ranges from 300 mm to 500 mm annually. Annual temperatures range from a minimum of 14 °C and a maximum of 34 °C. The region also experiences periodic ElNiño/Southern Oscillation (ENSO) phenomena which predispose it to epidemic RVF outbreaks [12]. The altitude of the study area varies between 18 m and 75 m above sea level with the coordinates of 1.5988°S and 40.5135°E. The area is inhabited mainly by pastoralists who engage in keeping livestock such as sheep, goats, cattle, camels, and donkeys, and migrate throughout the year in search of pastures and water. Vegetation in the area is predominantly shrubs and acacia bushes. Traps were set up in Sangailu, Ijara, Masalani, Korisa and Kotile communities of Ijara sub-County which have a lot of visible dambos in the landscape (shallow depressions that hold water during flooding and serve as breeding sites for flood water *Aedes*).

Kilifi County is relatively wet with two rainfall seasons; the short rains between October and December, and long monsoon rains between April and July, with an average annual rainfall of 950 mm. The annual temperatures range from a minimum of 21 °C and a maximum of 32 °C. The area lies 3.6333°S and 39.8500°E with an altitude of between 9 and 50 m. In this area, trapping was conducted in an urban setting with largely modified topography and vegetation providing numerous breeding sites for *Ae. aegypti*.

### Ethical statement

In Garissa, field trappings were conducted away from homesteads and on community land as authorized by area chiefs and community elders after explaining the purpose of the study to them. In Kilifi, informed consent was obtained from the persons in charge of public sites or heads of the homesteads where the studies were conducted as well as the area chiefs.

### Chemicals used

The lures tested in this study included commercial synthetic linalool oxide, commercial BioGent (BG) lure (a 3-component blend comprising ammonia, lactic acid and caproic acid developed for *Aedes aegypti*), and HONAD (a 4-component blend comprising heptanal, octanal, nonanal and decanal, an animal-based lure developed at the International Centre of Insect Physiology and Ecology (*icipe*) in Nairobi for RVF vectors). The composition of HONAD is 2 mg/ml heptanal, 0.5 mg/ml octanal, 0.1 mg/ml nonanal and 0.1 mg/ml decanal [24].

The synthetic standards of the following compounds were used: linalool oxide (Aldrich, mixture of stereoisomers with furanoid form, 99.5 % and 0.5 % pyranoid form), heptanal (Sigma-Aldrich, 98 %), octanal (Sigma-Aldrich, 98 %), nonanal (Sigma-Aldrich, 98 %), and decanal (Sigma-Aldrich, 98 %). Both LO and HONAD were released from a rubber septa.

### Optimization of field attractive doses of linalool oxide and determination of the release rate

Linalool oxide was tested at the concentration reported in our previous work (2 ng/μl) [32] including two ten-fold higher concentrations (20 ng/μl and 200 ng/μl) to find out if the threshold of odor response differs among the mosquito species. This was carried out at both Garissa and Kilifi field sites.

In Garissa, initial assessment of the dose responses was carried out in Sangailu involving three unlit CDC traps each baited with one of the three LO doses and CO_2_ and randomly placed in the vegetation around dambos and away from homesteads at 40 m inter-trap distance. This was replicated three times with each replicate set at a new location daily. The traps were activated at 18:00 hr and retrieved 06:00 hr the following morning. The trapped mosquitoes were knocked down using dry ice, sorted, counted and then placed in eppendorf tubes and preserved in liquid nitrogen for transport to the laboratory at *icipe* in Nairobi. Once at *icipe* the samples were stored at −80 °C until identification using morphological keys [36, 37].

Similarly, BG sentinel traps baited with each of the LO doses and CO_2_ was used to optimize for the most attractive dose for dengue vectors in Kilifi. Traps were placed at a distance of 40 m apart around three different locations (two breeding sites comprising abandoned tires and fish ponds which had *Ae. aegypti* larvae and one next to homestead with no obvious breeding sites) for two alternate days and one night. The daytime trapping was carried out from 06:00 – 18:00 hr while the night trapping was done from 18:00 – 06:00 hr. The trap captures were emptied and counted at the end of each trapping.

Release rate studies were carried out at the *icipe* Duduville campus in Nairobi (1.22°S, 36.88°E; ≈ 1,600 m above sea level) with temperature variations between 12 and 28 °C and humidity of 60–70 %. The release rate of LO at the optimal dose (20 ng/μl) over 12 hr period was determined by applying 100 μl of the LO solution on a rubber septa, allowing the solvent to evaporate completely in a fume chamber before exposing the rubber septa outside. Volatiles were collected from the rubber septa in a 40 ml quickfit chamber (ARS, Gainesville, FL, USA®) and passing air over it at a flow rate of 260 ml/min into an adsorbent Super-Q trap for 1 hr. Volatiles were collected after every three hour-interval over a 12 hr period as follows: 1 hr, 3 hr, 6 hr, 9 hr and 12 hr; with the rubber septa re-exposed outside after every collection. The Super-Q trap was eluted with dichloromethane and analyzed on coupled gas chromatography–mass spectrometry (GC/MS). The GC/MS analysis was carried out in the splitless injection mode using an Agilent Technologies 7890 gas chromatograph coupled to a 5975C inert XL EI/CI mass spectrometer (EI, 70 eV, Agilent, Palo Alto, California, USA) equipped with an HP-5 column (30 m × 0.25 mm ID × 0.25 μm film thickness, Agilent, Palo Alto, California, USA). Helium was used as the carrier gas at a flow rate of 1.2 ml/min. The oven temperature was held at 35 °C for 3 min, then programmed to increase at 10 °C/min to 280 °C and maintained at this temperature for 5 min. Three replicates were carried out at each time interval and the average peak areas for the two stereoisomers of LO ((*Z*)*-* and (*E*)-linalool oxide (furanoid form)) used to quantify release rates against an external calibration with synthetic LO.

## Study design

### Effectiveness of LO in trapping Rift Valley fever vectors

#### Selection of a suitable trap type

In a preliminary study, the performance of CDC light trap and BG sentinel trap were compared in terms of captures to determine the best trap type for RVF vectors. Three unlit CDC traps and three BG sentinel traps separately baited with LO + CO_2_, HONAD + CO_2_ and BG lure + CO_2_ were randomly set around vegetation with water-containing dambos away from homesteads. With an inter-trap distance of 40 m, the experiment was replicated five times. Based on consistent higher captures compared to BG sentinel trap (Additional file 1: Figure S1), CDC light trap was selected for subsequent evaluation of the effectiveness of the different lures in trapping RVF vectors.

#### Evaluation of lures

Two sets of experiments were carried out. In the first experiment, 5 treatments comprising unlit CDC traps each baited singly with CO_2_, LO, HONAD, BG lure and lit CDC trap without CO_2_ were compared. A total of five replications were carried out in a Completely Randomized experimental design with each replicate set in a new location in either Sangailu or Kotile sites. In the second experimental setup, similar 5 sets of treatments comprising unlit CDC traps each baited with CO_2,_ LO + CO_2_, HONAD + CO_2_, BG lure + CO_2_ and a lit CDC trap + CO_2_. Completely Randomized study design was conducted over five different sites (Sangailu, Ijara, Masalani, Korisa, and Kotile) located approximately 30–100 km apart. At each site, traps were set up following a Completely Randomized experimental design as described above. The study was carried out over 12 nights with each night treated as a replicate. Each site was sampled at least twice and all the treatments rotated through all the sites. In both experiments, traps were activated shortly after sunset (18:00 hrs) and removed in the morning (06:00 hrs). The synthetic carbon dioxide in the form of dry ice was released from an Igloos thermos container (2 L; John W Hock, Gainesville, FL, USA). Trapped mosquitoes were knocked down, preserved and transported to *icipe* as described above. Once at *icipe* the samples were stored at −80 °C until identification using morphological keys [36, 37] and the number of target species counted.

### Effectiveness of LO in trapping dengue vectors

Mosquito trapping was done within the urban centre in Kilifi. Two sets of experiments were carried out. In the first experiment, three treatments comprising BG sentinel traps each baited with CO_2_, LO and BG lure were compared at six sites; two near productive breeding sites comprising abandoned tires or fish ponds (sites with *Ae. agypti* larvae), two in vegetation, and two around homesteads. A 3 × 6 Latin Square study design was used comprising six days and six nights. The traps were rotated through all the sites with each treatment replicated twice in each of the six sites day and night, giving a total of 12 replicates. In the second experiment, three treatments comprising BG sentinel traps each baited with CO_2_, LO + CO_2_ and BG lure + CO_2_ were compared in a 3 × 6 Latin Square experimental design as described above with a total of 12 replicates. Day time mosquito trapping was done between 06:00 hrs and 18:00 hrs while night time trapping was done between 18:00 hrs and 06:00 hrs the following day. The carbon dioxide was dispensed as described earlier. Trap collections were removed at 06:00 hr and 18:00 hrs every day, knocked down, counted and similarly preserved and transported as described earlier. Once at *icipe* the samples were stored at −80 °C until identification using morphological keys [36, 37].

### Statistical analysis

The difference in the release rates of (*Z*)- and (*E*)- Linalool oxide (furanoid) over time was detected by one-way ANOVA after reciprocal transformation of release rates. The proportions of total number of mosquitoes trapped by each dose of LO were subjected to Chi square. Similarly, trap capture between CDC and BG sentinel traps to determine the best trap type for RVF vectors was compared using Chi square. To determine the effectiveness of the different lures in trapping disease vectors, the numbers of mosquitoes per treatment were first fitted with general linear model (GLM) with Poisson distribution and then negative-binomial error structure and log link in case of over dispersion as described by White and Bennetts [38] using R 2.15.1 software [39]. For RVF vectors, trap treatment (lure) was modeled as factors, with BG lure and BG lure + CO_2_ serving as reference for traps without and with CO_2_, respectively. Trap captures in the presence and absence of CO_2_ was compared using Chi square. In the case of dengue vectors, trap treatment and time of the day, were modeled as factors, with BG lure or BG lure + CO_2_ serving as reference for traps without and with CO_2_, respectively. Similarly, trap captures in the presence and absence of CO_2_ was compared using Chi square. The incidence rate ratios (IRR), a measure of the likelihood that mosquito species chose treatments other than the reference treatment (traps baited with BG lure or BG lure + CO_2_) and their *P*-values were estimated. The IRR for the reference is 1 (unity) and values above this indicates better performance and values below under performance of the treatments relative to the control. Given the high number of male mosquitoes caught in the traps for *Ae. aegypti*, captures were compared for both sexes. In addition, day and night captures of *Ae. aegypti* were compared using Chi square goodness-of-fit test. All statistical analyses were done at 95 % confidence interval.

## Results

### Optimal field attractive dose of LO and its release rates

**Fig. 1:**
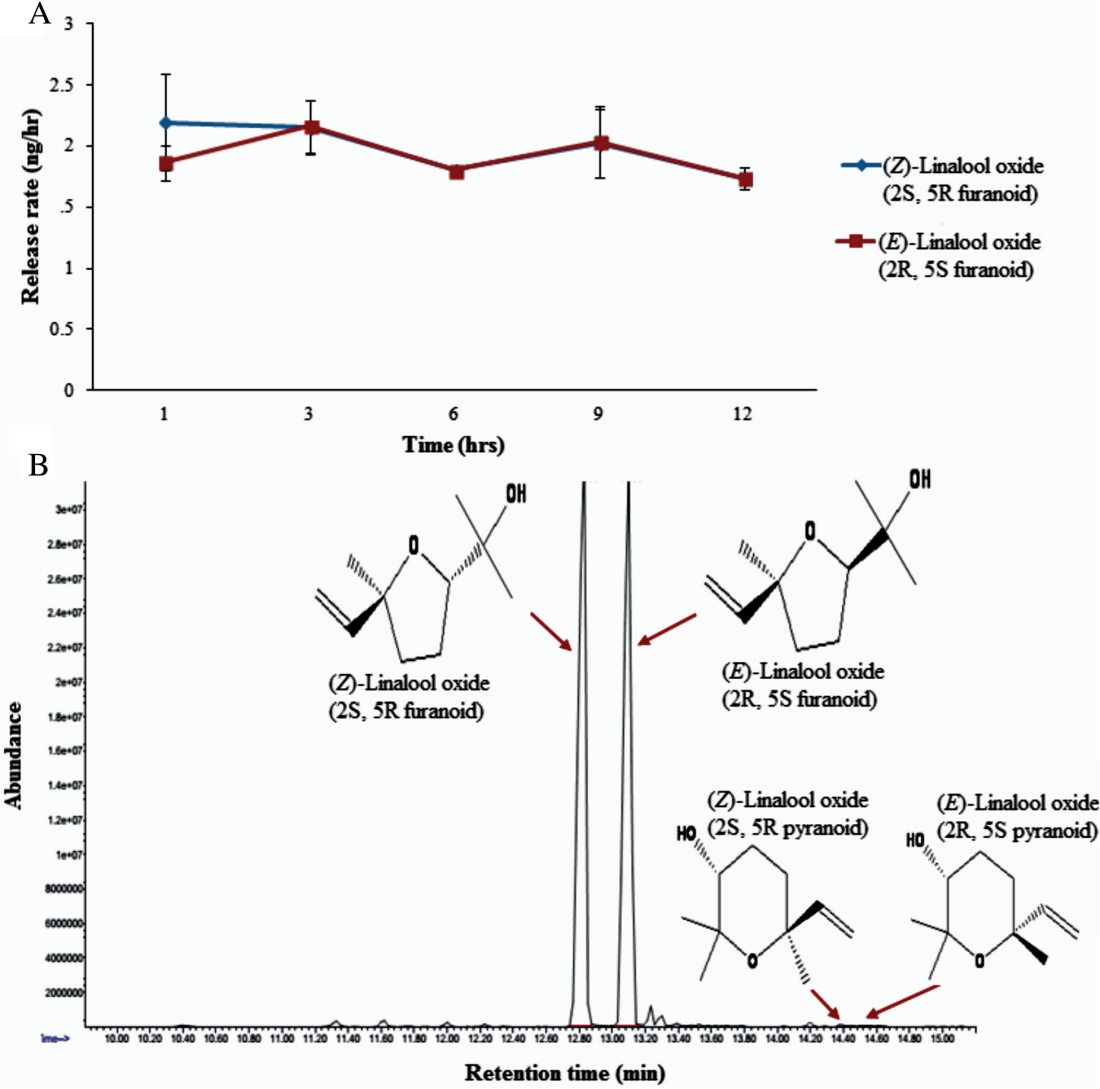
Release rates of the optimal dose of LO and its structural isomers: **a** release rate of LO at dose of 20 ng/μl collected over 12 hr period, **b** GC/MS chromatogram showing their retention time and structures of the four isomers of LO

We determined that for both RVF and dengue vectors the most attractive dose was 20 ng/μl, which recorded the highest mosquito trap captures. However, a significant difference between the trap captures for the three doses was only evident for *Aedes agypti* (*P* < 0.001) but not for *Ae. mcintoshi* and *Ae. ochraceus* (Table 1). The average release rate at this dose was 1.98 ng/hr and 1.92 ng/hr for (*Z*)*-* and (*E*)-linalool oxide (furanoid), respectively, with no difference in the release rates of both isomers over the 12 hr period (F_(4,10)_ = 0.746, *P* = 0.582 and F_(4,10)_ = 0.965, *P* = 0.468, respectively) (Fig. 1a). GC/MS analysis further revealed that the commercial synthetic standard of LO used also contained trace amounts of the pyranoid form of (*Z*)*-* and (*E*)-linalool oxide (Fig. 1b).

**Table 1:** Trap captures of Rift Valley fever and dengue vectors to different doses of Linalool oxide

Mosquito species	2 ng/μl	20 ng/μl	200 ng/μl	*P*-value
*Aedes mcintoshi*	19	28	24	0.276
*Aedes ochraceus*	11	21	15	0.088
*Aedes aegypti*	297	391	346	<0.001

The values represent the total number of mosquitoes caught. Unlit CDC traps were used in trapping *Ae. mcintoshi* and *Ae. ochraceus*, while BG sentinel trap was used in trapping *Ae. aegypti*

### Linalool oxide effective in trapping Rift Valley fever vectors

In the first experiment, low numbers of *Ae. mcintoshi* and *Ae. ochraceus* were caught in the absence of CO_2_ (*Ae. mcintoshi*: LO = 4, HONAD = 1, BG lure = 4, lit CDC trap =5; *Ae. ochraceus*: LO = 5, HONAD = 7, BG lure = 2, lit CDC trap =6) with no significant difference in the mosquito captures between the traps baited with these lures (*P* = 0.33). In the second experiment, a total of 267 *Ae. mcintoshi* and 141 *Ae. ochraceus* were caught in 12 replicate trials. The lit CDC traps + CO_2_ caught significantly more *Ae. mcintoshi* than the unlit CDC traps baited with BG lure + CO_2_ (IRR = 2.1, *P* < 0.001), while the unlit CDC trap + CO_2_ caught the least number of this species (IRR = 0.1, *P* < 0.001, Table 2). HONAD + CO_2_ and LO + CO_2_ did not differ significantly from the BG lure + CO_2_ in trapping *Ae. mcintoshi* (IRR = 1.1 and 1.3, respectively, Table 2). Similarly, the lit CDC trap + CO_2_ caught significantly a higher number of *Ae. ochraceus* than the BG lure (IRR = 2.6, *P* < 0.001), with the unlit CDC trap + CO_2_ catching the least number (IRR = 0.4, *P* < 0.05, Table 2). Again, trap captures with HONAD + CO_2_ and LO + CO_2_ did not differ significantly from that captured by the BG lure + CO_2_ (IRR = 0.8 and 1.1, respectively, Table 2).

**Table 2:** Trap captures of Rift Valley fever vectors captured by CDC trap baited with different lures

Mosquito species	Lure	Sample size	Mean ± SEM	*P-*value
*Aedes mcintoshi*	BG lure + CO_2_	47	3.92 ± 0.93	
	CO_2_	7	0.58 ± 0.19	<0.001
	LO + CO_2_	59	4.92 ± 1.56	0.24
	HONAD + CO_2_	53	4.42 ± 1.22	0.55
	Lit CDC + CO_2_	101	8.42 ± 1.05	<0.001
*Aedes ochraceus*	BG lure + CO_2_	24	2.00 ± 0.63	
	CO_2_	10	0.83 ± 0.27	<0.05
	LO + CO_2_	26	2.17 ± 0.91	0.78
	HONAD + CO_2_	19	1.58 ± 0.63	0.44
	Lit CDC + CO_2_	62	5.17 ± 1.14	<0.001

Lit CDC trap was not baited with any lure except CO_2_. Total number of replicates (N) = 12. *SEM* standard error of mean, *LO* linalool oxide, *BG* Biogent. BG lure + CO_2_ was used as reference and *P*-values for each treatment relative to it calculated

### Linalool oxide effective in trapping dengue vectors *Aedes aegypti*

In the first experiment, 628 females and 804 males of *Ae. aegypti* were caught. There was no significant difference between LO and BG lure in trapping female *Ae. aegypti* (IRR = 1, *P* = 0.89, Table 3), but LO trapped 1.4 fold more males than the BG lure (*P* < 0.01, Table 3). Traps baited with carbon dioxide alone were less attractive compared with those baited with the BG lure in trapping both male and female *Ae. aegypti* (IRR = 0.4, *P* < 0.001; and IRR = 0.3, *P* < 0.001, respectively, Table 3). When the lures were each combined with CO_2_, a total of 2087 female and 2415 male *Ae. aegypti* were caught. An additive effect on trap capture was observed (*P* < 0.001), with LO trapping significantly more males (IRR = 2.8, *P* < 0.001, Table 3) than the BG lure. The number of male and female *Ae. aegypti* caught were significantly higher during the day than at night (IRR = 3, *P* < 0.001 and IRR = 1.3,*P* < 0.001, respectively).

**Table 3:** Trap captures of dengue vectors (*Aedes aegypti*) captured by BG trap baited with different lures

Sex	Lure	Sample size	Mean ± SE	*P-*value
Females	BG lure	257	32.13 ± 9.48	
	LO	216	27.00 ± 6.43	0.89
	CO_2_	155	19.38 ± 4.49	<0.001
	BG lure + CO_2_	998	124.75 ± 53.54	
	LO + CO_2_	935	116.88 ± 66.03	0.15
	CO_2_	163	20.38 ± 3.94	<0.001
Males	BG lure	204	25.50 ± 9.87	
	LO	416	52.00 ± 17.08	<0.01
	CO_2_	174	21.75 ± 4.75	<0.001
	BG lure + CO_2_	710	88.75 ± 38.67	
	LO + CO_2_	1521	190.13 ± 72.41	<0.001
	CO_2_	177	22.13 ± 3.49	<0.001

Total number of replicates (N) = 12. *SEM* standard error of mean, *LO* linalool oxide, *BG* Biogent. BG lure or BG lure + CO_2_ was used as reference and *P*-values for the other non-CO2 and CO_2_ baited lures, respectively, calculated

## Discussion

In our previous study, we had demonstrated the high efficacy of LO alone and in combination with carbon dioxide in trapping the malaria vectors *An. gambiae* s.l. [32]. Our results showed that despite targeting different mosquito disease vectors, the three lures LO, BG and HONAD alone and in combination with carbon dioxide varied in their effectiveness in trapping RVF vectors *Ae. mcintochi* and *Ae. ochraceus* and the dengue vector *Ae. aegypti*, as previously found for the malaria vectors *An. gambiae* s.l. [32]. Notably, whereas LO alone was effective in trapping both sexes of *Ae. aegypti*, LO, BG lure and HONAD were only effective in trapping the two RVF vectors in the presence of CO_2_. The failure of the three lures to trap RVF vectors in the absence of CO_2_ perhaps explains the critical role played by this compound in host location and orientation by these vectors, which are highly zoophilic as opposed to the anthropophilic nature of *Ae. aegypti* and *An. gambiae* in agreement with previous studies [40].

Also, notably, besides a few laboratory-based bioassays to test the attractiveness of plant volatile extracts to *Ae. aegypti* [41, 42], this is the first field evidence of the potential of a plant-based compound in field trapping of RVF and dengue vectors. Linalool oxide compared favorably to the two vertebrate lures in trapping RVF and dengue vectors. This is particularly interesting given that LO is a single component lure capable of attracting a number of important mosquito species. Use of common chemical cues in host location has been demonstrated in several insect species. For instance, 1-octen-3-ol has been shown to play a key role in host location by several blood feeding insects including tsetse flies, stable flies, culicoides and mosquitoes [43–45]. These findings highlight the potential use of a single- or multi-component plant-based lure for mosquito vector surveillance and control.

The fact that CO_2_-baited lit CDC traps performed better than CO_2_-baited unlit CDC traps baited with each of the three lures for RVF vectors, suggests the importance of visual cues in the behavior of these mosquitoes. As such, the interaction of visual and chemical cues could be exploited further for effective monitoring of these vectors. Therefore, with further development and formulations, linalool oxide and perhaps other yet to be identified plant odors may hold promise as possible plant-based alternatives to CO_2_ depending on the target mosquito species.

Furthermore, the presence of male *Ae. aegypti* in traps baited with LO is interesting. With the development of sterile insect technique as a population reduction tool for mosquito control, there has been a need to improve field based lures to target the male segment of the mosquito population for purposes of evaluating competency and survival of sterile males as compared to wild ones [46]. Plant-based lures have been suggested to hold potential in targeting this segment of the population [28, 30]. Therefore, our finding here suggests the potential of a phytochemical in trapping both male and female mosquito disease vectors.

LO is a chiral compound and the commercial synthetic standard used in our study as shown by our GC/MS analysis consisted of tentatively identified racemic mixture of four enantiomers, (2R,5S)-(*E*)-furanoid, (2S,5R)-(*Z*)-furanoid, (2R, 5S)-(*E*)-pyranoid and (2S, 5R)-(*Z*)-pyranoid. Therefore, it is likely that the differential efficacy of LO in trapping the different mosquito disease vectors and sexes can be attributed to the different enantiomeric forms of this compound, which needs further investigation. Interestingly, differential response of insects to different stereoisomers of certain semiochemicals has been reported. For example, the (1S,2’S) form of 1-[3-cyclohexen-1-ylcarbonyl]-2-methylpiperidine was found to be two-fold more repellent against *Ae. aegypti* than the (1R,2’S) form [47]. Similarly, *Manduca sexta* was shown to have a higher preference for plants producing (+)-linalool compared to those producing (−)-linalool [48]. Hence the present study provides a basis for further investigation into the potential of phytochemicals, especially those with chiral centres to address specificity in responses of different mosquito disease vectors to stereoisomers of these compounds.

## Conclusion

We document the performance of LO in trapping the RVF vectors, *Ae. mcintoshi* and *Ae. ochraceus*, which compared favorably with the BG lure and HONAD, but better than the BG lure for female *Ae. aegypti*, the major vector of dengue. In the absence of carbon dioxide, this compound performed dismally in trapping RVF vectors but was comparable to the BG lure for trapping dengue vectors. However, linalool oxide was superior to the BG lure in trapping male *Ae. aegypti* in the presence or absence of carbon dioxide. These results highlight the potential of LO as a generalist plant-based lure for mosquito disease vectors, with room for further development to obtain a potent phytochemical attractant for the management of these vectors.

## Additional file

Additional file 1: Figure S1. Comparision of unlit CDC trap and BG sentinel trap in trapping Rift Valley fever vectors. *N* = 5, chi square was used to analyze count data. Bars capped with ** are significantly different at 0.01. (PNG 10 kb)

## Abbreviations

RVF: Rift Valley fever
LO: linalool oxide
BG: BioGent
HONAD: heptanal, octanal, nonanal and decanal
KEMRI: Kenya Medical Research Institute
GLM: generalized linear model
SIDA: Swedish International Development Cooperation Authority
