# Supplementary figures and images for: Linalool oxide: generalist plant based lure for mosquito disease vectors
Source: Parasit Vectors. 2015 Nov 9;8:581. doi: 10.1186/s13071-015-1184-8 (PMC4640417; doi:10.1186/s13071-015-1184-8)

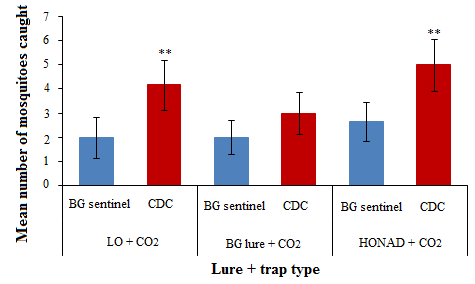

Supplement: Additional file 1: Figure S1. — Comparision of unlit CDC trap and BG sentinel trap in trapping Rift Valley fever vectors. N = 5, chi square was used to analyze count data. Bars capped with ** are significantly different at 0.01. (PNG 10 kb) [file 13071_2015_1184_MOESM1_ESM.png]
